# An aromatic imidazoline derived from chloroquinoline triggers cell cycle arrest and inhibits with high selectivity the *Trypanosoma cruzi* mammalian host-cells infection

**DOI:** 10.1371/journal.pntd.0009994

**Published:** 2021-11-29

**Authors:** Roberto I. Cuevas-Hernández, Richard M. B. M. Girard, Luka Krstulović, Miroslav Bajić, Ariel Mariano Silber

**Affiliations:** 1 Laboratory of Biochemistry of Tryps, Department of Parasitology, Institute of Biomedical Sciences, University of São Paulo, São Paulo, SP, Brazil; 2 Department of Chemistry and Biochemistry, Faculty of Veterinary Medicine, University of Zagreb, Zagreb, Croatia; Universidade Federal de São Paulo, BRAZIL

## Abstract

*Trypanosoma cruzi* is a hemoflagellated parasite causing Chagas disease, which affects 6–8 million people in the Americas. More than one hundred years after the description of this disease, the available drugs for treating the *T*. *cruzi* infection remain largely unsatisfactory. Chloroquinoline and arylamidine moieties are separately found in various compounds reported for their anti-trypanosoma activities. In this work we evaluate the anti-*T*. *cruzi* activity of a collection of 26 “chimeric” molecules combining choroquinoline and amidine structures. In a first screening using epimastigote forms of the parasite as a proxy for the clinically relevant stages, we selected the compound 7-chloro-4-[4-(4,5-dihydro-1H-imidazol-2-yl)phenoxy]quinoline (named here as **A6**) that performed better as an anti-*T*. *cruzi* compound (IC_50_ of 2.2 ± 0.3 μM) and showed a low toxicity for the mammalian cell CHO-K_1_ (CC_50_ of 137.9 ± 17.3 μM). We initially investigated the mechanism of death associated to the selected compound. The **A6** did not trigger phosphatidylserine exposure or plasma membrane permeabilization. Further investigation led us to observe that under short-term incubations (until 6 hours), no alterations of mitochondrial function were observed. However, at longer incubation times (4 days), **A6** was able to decrease the intracellular Ca^2+^, to diminish the intracellular ATP levels, and to collapse mitochondrial inner membrane potential. After analysing the cell cycle, we found as well that **A6** produced an arrest in the S phase that impairs the parasite proliferation. Finally, **A6** was effective against the infective forms of the parasite during the infection of the mammalian host cells at a nanomolar concentration (IC_50(tryps)_ = 26.7 ± 3.7 nM), exhibiting a selectivity index (*SI*) of 5,170. Our data suggest that **A6** is a promising hit against *T*. *cruzi*.

## Author summary

Chagas disease (CD) is caused by *Trypanosoma cruzi*, a homoflagellate parasite that belongs to the group of kinetoplastids. This disease is endemic to the Americas, however, the migrations have spread CD to other continents. An estimated that more than 10,000 die each year due to infection by *T*. *cruzi*, 8 million people are infected and therefore more than 25 million people are at risk of acquiring the disease. Treatments to CD are not very efficient, mainly in the chronic phase of the disease and cause adverse effects due to the toxicity of drugs currently in use. Therefore, there is an urgent need for new anti-*T*. *cruzi* drugs. In this work we evaluate the anti-*T*. *cruzi* capacity of a series of compounds derived from 7-chloroquinolines. We study in more detail the mode of action of A6, an aromatic imizoline derivative that showed a potent effect on the intracellular forms of the parasite (IC_50(tryps)_ = 26.7 ± 3.7 nM) and a high selectivity index (*SI* = 5,170). A6 could be a promising alternative for the development of a new efficient treatment against *T*. *cruzi*.

## Introduction

Chagas disease which is endemic from southern USA to southern Argentina and Chile is caused by *Trypanosoma cruzi*, which, according to the World Health Organization (WHO) about 8 million people are infected worldwide [1]. *T*. *cruzi* belongs to the group of the kinetoplastid hemoflagellated parasites, which have several unique characteristics. Among them, and relevant to this work, these parasites have one mitochondrion per cell with its DNA (approximately 30% of the total DNA in the cell) organized in a network of 20–30 maxicircles (of approximately 20 kbp) and some 20–30 thousand minicircles (of approximately 1 kbp). This DNA is a main constituent of a structure denominated kinetoplast (thus, it is denominated kinetoplastid DNA or kDNA) which is crucial to encode some of the mitochondrial proteins responsible for the functionality of this organelle [2,3].

*T*. *cruzi* is present in a variety of mammalian reservoirs, and transmission to (and among) humans occurs mostly through the infected triatomine insects during their blood feeding. Alternatively, inter-human transmission can occur as well through other routes, such as organ transplants, transfusions and ingestion of contaminated foodstuffs [4]. *T*. *cruzi* infection initiates presenting an acute phase that lasts for 2–8 weeks, characterized by a prominent parasitemia. In most of cases the acute phase is asymptomatic or present mild unspecific symptoms (such as fever, and headaches). If not treated, the disease evolves to a chronic condition that can be either symptomatic (30–40% of patients) or asymptomatic (70% of patients) [4]. Symptoms, when present, can appear from weeks to decades after the infection, and are associated with the clinical manifestations. Among them, the most relevant are the cardiac form and the digestive form of the disease.

The only drugs currently licensed for the treatment of Chagas disease are two nitroheterocyclic compounds introduced 50 years ago into clinical practice: benznidazole and nifurtimox. However, they are far away of being satisfactory due to the serious concerns they raise such as limited efficacy, especially in the chronic phase and adverse effects due to their toxicity, leading patients to abandon the treatment [5]. In this context, there is an urgent need to identify and develop new drugs against *T*. *cruzi*.

**Fig 1:**
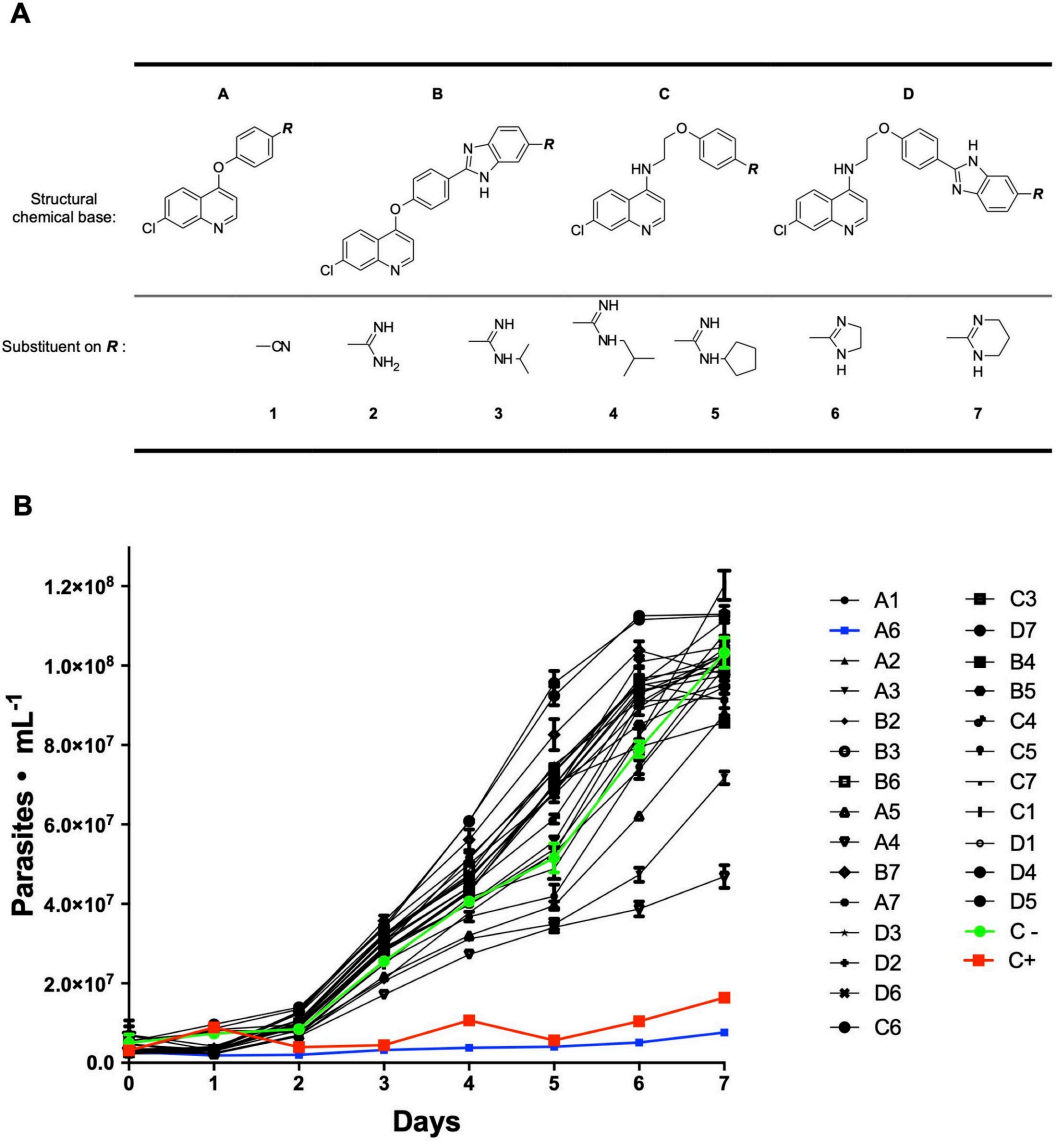
Screening assay for selection of compounds able to inhibit the proliferation of epimastigote forms of *T*. *cruzi*. **A)** Chemical structure of the tested compounds. **B)** Proliferation curves of epimastigotes of *T*. *cruzi* in the presence of 5 μM for each compound. Positive control (C+) for the inhibition of proliferation corresponds to curves obtained in the presence of 20 μM benznidazole, and negative controls correspond to curves obtained in the presence of the solvent (DMSO). Each experiment was run in quadruplicates in three independent experiments and values correspond to mean ± SEM.

Chloroquine, a 7-chloro-4-aminoquinoline derivative, has been used as an anti- malaria agent, since 1940s. This class of compound has shown a broad range of activities of pharmacological interest against *Leishmania spp*. [6,7], *T*. *cruzi* [8,9], bacteria, viruses, fungi [10,11] and tumors [12,13]. Several mechanisms of action have been proposed such as alkalinisation of phagolysosomes for intracellular bacteria and fungi [10], interference in the heme metabolism for anti-parasitic activities [9,10] or complex formation with DNA resulting in defects in the synthesis and repair of this macromolecule [14,15]. On the other hand compounds containing an amidine group have shown also interesting effects as anti-protozoal [16–18], anti-viral [19], anti-bacterial [20] and antitumoral [21]. Amidines (diamidines among them) mechanisms of actions are not completely understood, however it has been shown that this class of compound usually has a high affinity for DNA, particularly for the minor groove of AT-rich regions [22]. In fact, several studies demonstrated that the aromatic diamidines can accumulate and bind preferentially the kDNA, an AT rich regions DNA, leading to the interruption of the cell cycle [18,23]. Additionally, these derivatives can affect several mitochondrial functions such as ATP production or mitochondrial membrane potential maintenance [18]. Noteworthy, 7-chloro-4-aminoquinoline and amidines derivatives are found separately in a large number of pharmacological compounds. However, very few studies have reported the biological activity of “chimeric” compounds, that is, molecules in which different chemical groups combine in a single molecule. The present study evaluated the anti *T*. *cruzi* activity *in vitro* of 26 of these “chimeras” obtained by the conjugation of 7-chloroquinoline and arylamidine moieties (**Fig 1A**) [24].

## Materials and methods

### Reagents

All chemicals, reagents and solvents, were purchased from Sigma-Aldrich (St. Louis, MO, USA). Probes, such as Fluo-4 AM and Annexin V-FITC were purchased from Invitrogen (Eugene, Oregon, USA). Culture media and foetal calf serum (FCS) were purchased from Cultilab (Campinas, SP, Brazil).

### Tested compounds

The collection of tested molecules were obtained as previously described [24]. Described amidine compounds were obtained as di- or tri-hydrochloride salts. Due to its profile in the initial assays, we chose 7-chloro-4-[4-(4,5-dihydro-1H-imidazol-2-yl)phenoxy]quinoline) (named here as A6) for a refined evaluation of its anti-*T*. *cruzi* activity.

### Cells and parasite cultures

*T*. *cruzi* epimastigotes (CL strain clone 14) were maintained in exponential proliferation by subculturing the parasites every 48 h in Liver Infusion Tryptose (LIT) medium at 28°C [25]. The Chinese Hamster Ovary cell line CHO-K_1_ was cultivated in RPMI-1640 medium supplemented with 10% heat-inactivated Fetal Calf Serum (FCS), 0.15% (w/v) NaHCO_3_, 100 units/mL penicillin and 100 mg/mL streptomycin at 37°C in a humidified atmosphere containing 5% CO_2_. Trypomastigotes were obtained by infection in CHO-K_1_ cells with trypomastigotes as previously described [18]. Infected cells were maintained at 37°C in the presence of 10% FCS. After 24 h, the cells were maintained at 33°C and 2% FCS. Trypomastigotes were collected from the extracellular medium five days after infection.

### *In vitro* inhibition of proliferation assays on epimastigotes

The cell density of exponentially proliferating epimastigotes (approximately 5×10^7^ parasites/mL) was adjusted to 2.5×10^6^ cells/mL. The parasites were then transferred into 96-well plates (200 μL/well). Epimastigote proliferation was measured as previously reported, by reading the optical density (OD) at 620 nm every 24 h during 8 days (which allowed us to have readings through the exponential and stationary phases) [26]. The OD values were converted to cell density values (cells per millilitre) by using a calibration curve obtained by measuring the OD values at 620 nm of parasite suspensions at different known densities [27]. The half-maximal inhibitory concentrations (IC_50_) were determined from cell density data obtained at the 4^th^ proliferation day, which corresponded to the mid exponential proliferation phase. Data were analysed by a non-linear regression to a sigmoidal dose-response curve using GraphPad Prism v.6. DMSO-disolved benznidazole (final concentration of 20 μM) and untreated parasites grown in the presence of the same volume of DMSO used for the benzonidazole treatment, were used as positive and negative controls, respectively. The compounds were evaluated in quadruplicate in each experiment, and the results correspond to three independent experiments.

### The effect of the compounds on mammalian cell viability

The viability of CHO-K_1_ cells was evaluated by assessing the irreversible reduction of resazurin (7-hydroxy-3H-phenoxazine-3-one 10-oxide) to resorufin (7-hydroxy-3H-phenoxazin-3-one), a redox fluorimetric indicator. The resazurin is usually considered as a reliable indicator of viability by active metabolism in cell cultures [28]. Briefly, CHO-K_1_ cells (1.0x10^5^ cells/well) in 100 μL of RPMI medium supplemented with FCS (10%) were seeded in 96-well plates with or without (control) different concentrations of the tested compounds. After 48 h, the cell viability was determined by the rezarzurin assay, the cells were incubated in the presence of 0.125 μg/μl resazurin for 3 h at 37°C in the absence of light. The fluorescence signal was measured in a Spectra Max M3 fluorometer (Molecular Devices) at λ_exc_ 560 nm and λ_em_ 590 nm. The IC_50_ values were determined by fitting a sigmoidal dose-response curve to the data using GraphPad Prism v.6. Each assay was developed in triplicate and the results correspond to the mean of three independent experiments.

### Analysis of phosphatidylserine exposure

Epimastigotes (2.5 x10^6^ cell/mL) were incubated for four days in the presence or absence (control) of 2.2 μM and 4.4 μM **A6** (concentrations corresponding to 1 or 2 times the IC_50_, respectively). To determine the exposure of phosphatidyl serine, the cells (1.0x10^6^) were labelled with propidium iodide (PI) and Annexin-V FITC (Molecular Probes) according to the manufacturer’s instructions. As positive controls for plasma membrane permeabilization and extracellular exposure of phosphatidylserine, the parasites were treated with 150 μM digitonin for 30 min [29]. The cells were analysed by flow cytometry on a BD Accuri™ C6 Plus, each condition was run in three biological independent replicas with 10,000 events collected and analysed using BD CSampler Plus Software (v 1.0.27.1) and FlowJo software (v07).

### Analysis of intracellular Ca^2+^ levels

Epimastigotes (2.5 x 10^6^ cell/mL) were incubated at different times for short-term (0, 1, 3 and 6 h) and long-term (four days) measurements of intracellular Ca^2+^ levels in the presence or absence (control) of 1.1 μM or 2.2 μM **A6** (approximately 0.5 x IC_50_ and 1 x IC_50_). Then the parasites (1.0x10^7^ cells) were washed with Phosphate Buffered Saline (PBS) and incubated with 5 μM Fluo-4 AM (Invitrogen) for one hour at 28° C, washed twice with HEPES glucose (50 mM HEPES, 116 mM NaCl, 5.4 mM KCl, 0.8 mM MgSO_4_, 5.5 mM glucose and 2 mM CaCl_2_, pH 7.4), resuspended in the same buffer and aliquoted into 96-well plates (2.5x10^7^ per well) [30]. Readings were performed on a Spectra Max I3 fluorometer, (Molecular Devices) at λ_exc_ 490 nm and λ_em_ 518 nm. Each assay was developed in triplicate and the results correspond to the mean of three independent experiments.

### Determination of *T*. *cruzi* intracellular ATP levels

Epimastigotes (2.5 x 10^6^ cell/mL) were incubated at different times for short-term (0, 1, 3 and 6 h) and long-term (four days) measurements of ATP levels in the presence or absence (control) of 1.1 μM and 2.2 μM **A6** (concentrations corresponding to approximately 0.5 x and 1 x IC_50_, respectively). Intracellular ATP levels were measured by using a bioluminescent assay kit according to the manufacturer’s instructions (Sigma-Aldrich). Briefly, 50 μL PBS was added to 100 μL cellular ATP-releasing reagent and added to a 50 μL suspension of 5.0x10^6^ parasites, treated or not treated (control). Light emission levels were measured on a Spectra Max I3 fluorometer at 570 nm [26]. Each assay was developed in triplicate and the results correspond to the mean of three independent experiments.

### Analysis of mitochondrial inner membrane potential (ΔΨ_m_)

Epimastigotes (2.5x10^6^ cell/mL) were incubated at different times for short-term (0, 1, 3 and 6 h) and long-term (four days) measurements of ΔΨ_m_ in the presence or absence (control) of 2.2 μM and 4.4 μM **A6** (concentrations corresponding to 1 or 2 times the IC_50_, respectively). For determining variations in ΔΨ_*m*_, cells were aliquoted in fractions at densities of 5.0x10^6^ cells/mL and the parasites were washed twice in PBS by centrifugation (2,700 x *g* for 5 min). The positive control was incubated for 15 min with 10 μM carbonyl cyanide-4-(trifluoromethoxy) phenylhydrazone (FCCP) in PBS. Then, all samples were centrifuged for 10 min at 2,700 x *g* and resuspended in PBS. The cells were labelled, or not for the unstained control, by the addition of 256 nM Rhodamine 123 (Rho123) for 20 min at 28°C. The cells were twice in washed with cytomix buffer (25 mM HEPES-KOH, 120 mM KCl, 0.15 mM CaCl_2_, 2 mM EDTA, 5 mM MgCl_2_, 10 mM K_2_HPO_4_/KH_2_PO_4_ buffer, pH 7.6, and 10 μM FCCP if indicated) and resuspended in 500 μL of the same buffer [31]. Changes in the cell’s fluorescence labelled with Rho123 were analysed by flow cytometry on a BD Accuri™ C6 Plus. Each condition was run in three biological independent replicas with 10,000 events collected and analysed using BD CSampler Plus Software (v 1.0.27.1) and FlowJo software (v07).

### Effect of A6 on the epimastigotes H_2_O_2_ production

To evaluate the effect of **A6** on H_2_O_2_ production rates, exponentially growing parasites (5x10^7^ cells per ml) were washed twice in PBS and resuspended in 125 mM saccharose, 65 mM KCl, 10mM HEPES, 1 mM MgCl_2_, 2.5mM K_2_HPO_4_, pH 7.2. The parasites were incubated with 5 μM Amplex red (AmR) and 0.01 μg/mL of Horse Radish Peroxidase (HRP) in the 2 ml chamber of a high-resolution oxygraph (OROBOROS, Oxygraph-2k, Innsbruck, Austria) equipped with a fluorometric measurement device. Then, 2.2 μM, 4.4 μM or 6.6 μM **A6** (1x, 2x, and 3x the IC_50_ concentrations) were sequentially added. H_2_O_2_ production was quantified by following the reduction of AmR which emits fluorescence in its reduced state as described in detail [32]. Experiments in each condition were run in three biological independent replicas and data were recorded and treated by using DatLab 7 software.

### DNA content and cell cycle analysis

Epimastigotes (2.5x10^6^ cells/mL) were incubated (or not, negative control) in the presence of 2.2 and 4.4 μM **A6** for four days. Then, the cells (1.0x10^7^ cells/mL) were collected by centrifugation (2,700 x *g* for 5 min), washed in PBS and fixed in 70% ethanol for 4 h. The parasites were washed twice in PBS and incubated with 10 μg/mL RNase A (Thermo Scientific) for 30 min at 37°C. To measure the DNA content, the cells were stained with 40 μg/mL propidium iodide (Molecular Probes/Invitrogen) and analysed by flow cytometry on a BD Accuri™ C6 Plus, with 50,000 events collected from three biological independent experiments [31]. Histograms (number of counts by FL2 area), scatter plots (side scatter [SSC] area by forward scatter [FSC] area) and gates for each cell cycle phase were analysed using BD CSampler Plus Software (v 1.0.27.1) and FlowJo software (v07). Cell cycle data were fitted by a model included in the FlowJo software (v07).

### Effect of A6 on amastigote replication and trypomastigote release

CHO-K_1_ cells (1.0 x 10^4^ per well) were seeded in 96-well plates in RPMI medium supplemented with 10% FCS at 37°C for 24 h. Then, the cells were incubated with 5.0x10^5^ trypomastigotes per well for 4 h. After this period, parasites in the supernatant were removed by washing the plates twice with PBS, and the cells were incubated overnight in RPMI medium supplemented with 10% FCS at 37°C in the presence of different concentrations of **A6** or left untreated (control) [27]. After 24 h, the plates were incubated at 33°C and RPMI 2% FCS with the same different **A6** concentrations to allow the parasites to complete the infection cycle. To measure the effect on amastigote replication, after 48 h the CHO-K_1_ cells and parasites were fixed with 4% paraformaldehyde and stained with Hoechst 33342. Images were taken by fluorescence microscopy. Cells, parasites, and infected cells were counted using ImageJ software. The infection index (percentage of infected cells × the number of parasites per cell) was calculated. The effect of **A6** on *T*. *cruzi* trypomastigotes release was determined on the fifth day post-infection, by counting the trypomastigotes released in the extracellular medium, using a Neubauer chamber. Each condition was assayed in three independent biological experiments.

### Data treatment and statistical analysis

Curve adjustments, regressions, and statistical analyses were performed with the GraphPad Prism 7 analysis tools. All assays were performed at least in biological triplicates. The specific details of the statistical analysis for each experiment are described in the corresponding figure legend. P values of less than 0.05 were considered statistically significant.

## Results

### Compound A6 affects the proliferation of *T*. *cruzi* epimastigotes

**Fig 2:**
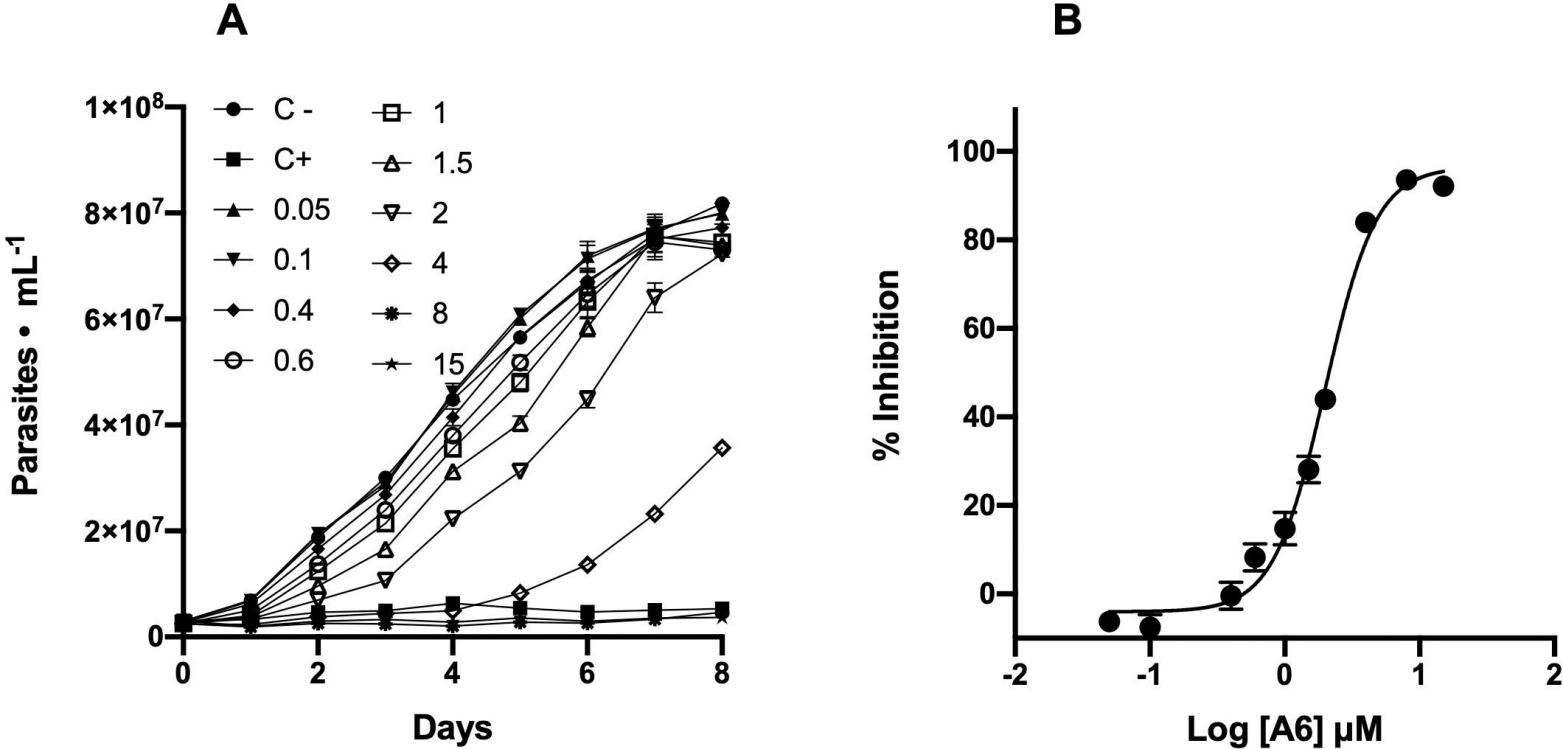
Effect of A6 on the proliferation of the epimastigotes of *T*. *cruzi*. **A)** Proliferation curves in the presence of different concentrations of **A6**, **B)** Dose-response curve. Values for proliferation were obtained from the proliferation curves shown in A at day 4th. Proliferation of the epimastigotes without treatment (negative control) was used to as a reference of 0% inhibition. All other values were obtained for the different concentrations of **A6** as indicated in Materials and Methods. An IC_50_ = 2.2 ± 0.3 μM was obtained by adjusting a nonlinear regression to the data. Results correspond to mean ± SEM of three independent experiments for each condition. Each curve in each independent experiment was made in quadruplicate.

To make an initial selection of active compounds against *T*. *cruzi* among the 26 7-chloroquinoline derivatives (**Fig 1A**), we initially incubated the parasites in the presence of each compound at a fixed concentration of 5 μM. For this, we followed the cell density during 8 days, and selected those compounds that were able to significantly inhibit the proliferation at the mid-exponential phase (4^th^ day). **A6** was the only compound in the collection that appreciably inhibited the increase of cell density by 90.8% when compared to untreated cultures (**Fig 1B and S1 Table**). Therefore, it was selected to measure its IC_50_ on epimastigote proliferation in order to assess its potential efficacy against *T*. *cruzi*. Compound **A6** exhibited a dose-dependent cells growth inhibition with IC_50_ value of 2.2 ± 0.3 μM (**Fig 2**).

### Cytotoxicity of A6 on mammalian cells

**Fig 3:**
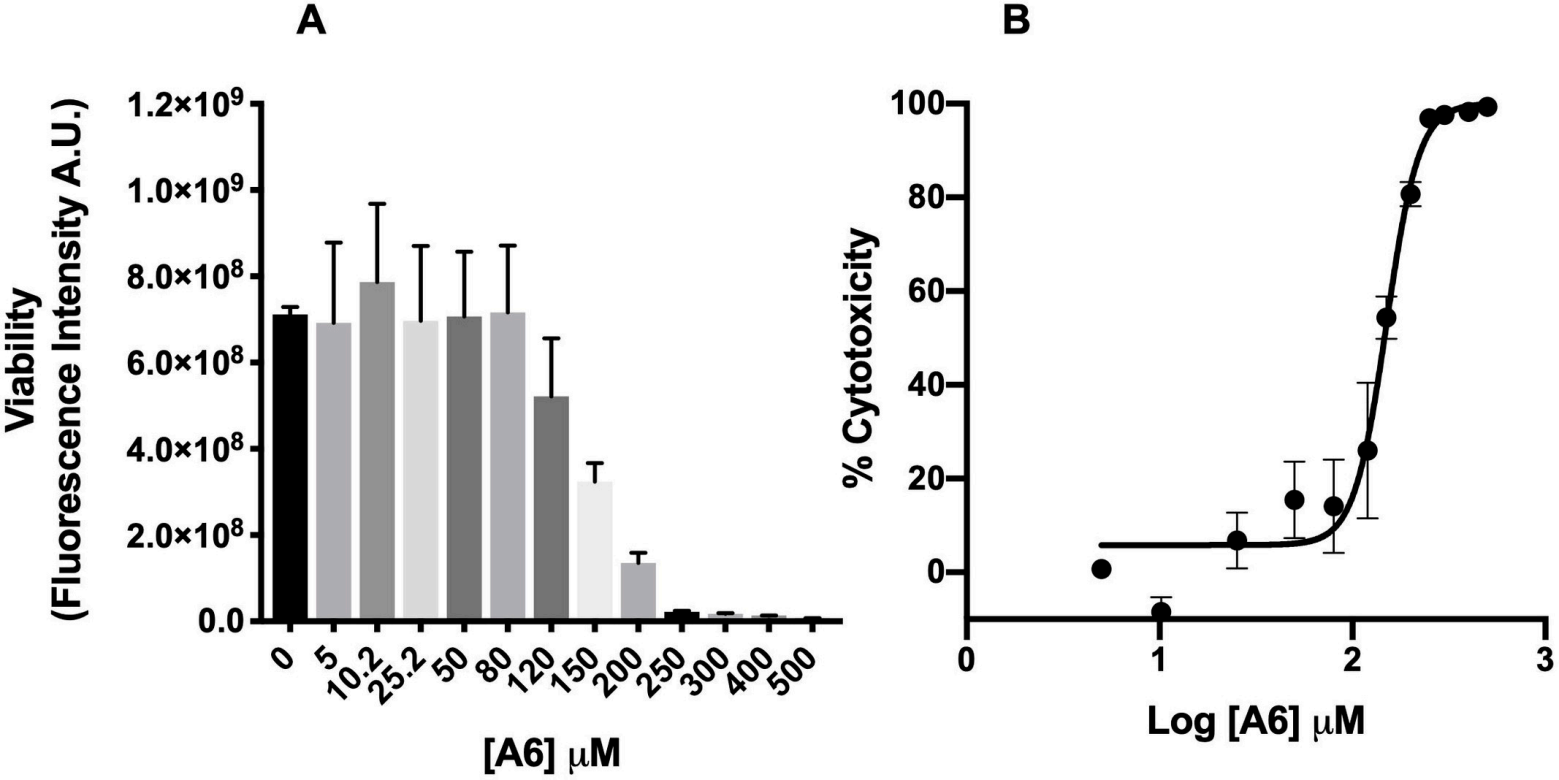
Effect of A6 on mammalian cells. The viability of CHO-K_1_ cells treated with different concentrations of A6 for 48 h was assessed by resazurin assay, and the inhibition of proliferation was expressed as a percentage. **A)** Cell viability of CHO-K_1_ cells measured as fluorescence intensity due to the production of resorufin, in the presence of different concentrations of **A6** (range: 0 to 500 μM). **B)** Dose—response curve for **A6**, obtained from data in A. A CC_50_ = 137.9 ± 17.3 μM was obtained by adjusting a nonlinear regression to the data. Results correspond to mean ± SEM of three independent experiments for each condition. Each curve in each independent experiment was made in triplicate.

To further evaluate **A6** as potential anti-*T*. *cruzi* agent, we measured, its toxicity in CHO-K_1_, used here as a model of a mammalian host cell. Briefly, CHO-K_1_ cells were cultured or not (control) in the presence of different concentrations of **A6** (between 0 and 500 μM) for 48h. The cytotoxicity was assessed using an assay based on the reduction of resazurin (**Fig 3A**), which assumes that the reductase activities in the treated cells with respect to untreated controls are reliable indicators of cell viability [33]. The cytotoxicity (CC_50_) was obtained by fitting a sigmoidal dose-response curves to the data. The concentration corresponding to a 50% decrease in the reductase activities was 137.9 ± 17.3 μM for **A6** (**Fig 3B**). These data allowed us to determine an initial selectivity index (*SI*; CC_50_/IC_50_) for the effect of **A6** on epimastigotes proliferation of 63. Based on these values, we selected **A6** to continue our investigation on the effect of the compound on different aspects of *T*. *cruzi* biology.

### A6 does not trigger membrane permeabilization and/or phosphatidylserine exposure

**Fig 4:**
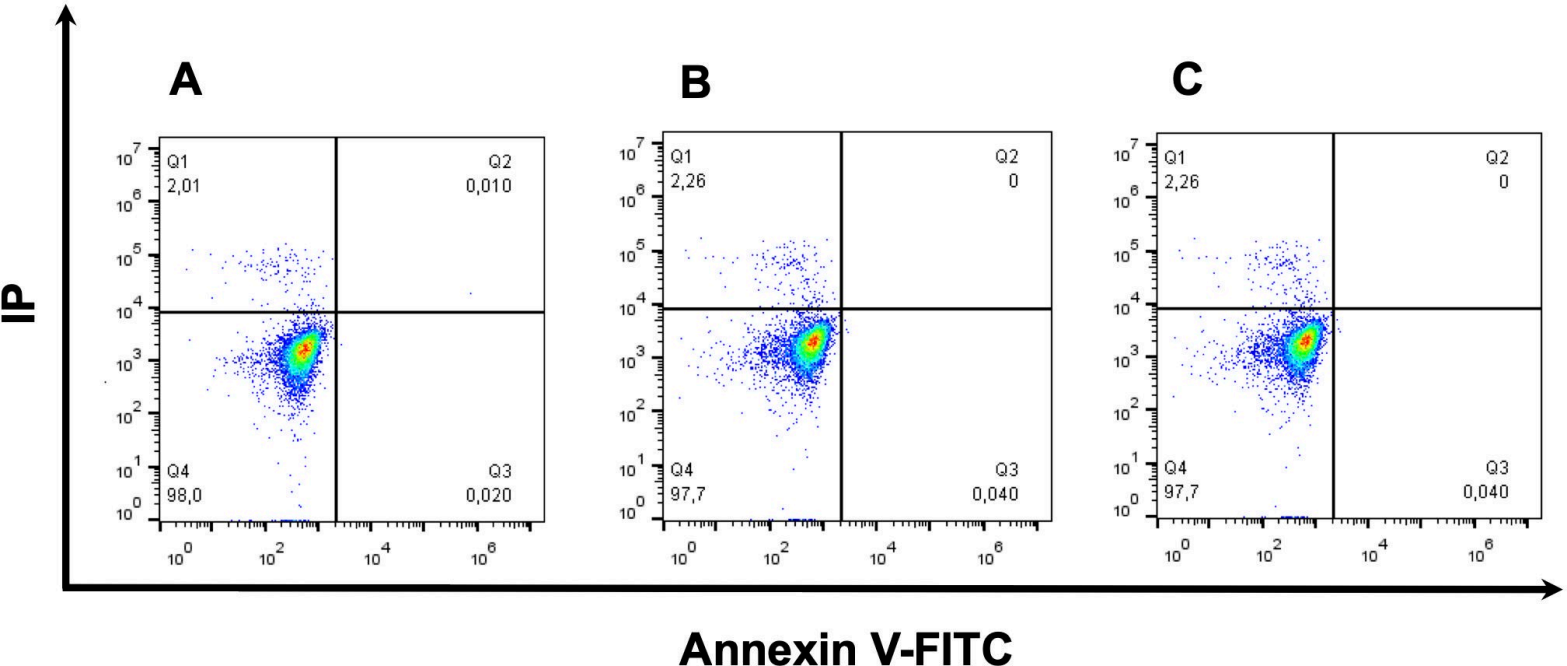
Cell death analysis of epimastigotes treated with A6. Cells were treated or not (control) with **A6** and simultaneously assessed for the extracellular exposure of phosphatidylserine (by using Annexin V) and plasma membrane integrity (by using propidium iodide labelling) followed by flow cytometry. Cells were treated or not with **A6** for four days. **A)** Non-treated parasites (control), **B)** parasites treated with 1 x IC_50_ (2.2 μM) **A6** and **C)** parasites treated with 2 x IC_50_ (4.4 μM) A6. Each experiment was run in three biological independent replica with 10,000 events being collected and analysed.

To explore the mechanism involved in the arrest of epimastigotes proliferation by **A6**, we initially evaluated its capacity to affect the plasma membrane functionality. For this, we evaluated **A6**-treated epimastigotes for plasma membrane integrity and for the exposure of phosphatidylserine in its external leaflet. The cells were then analysed by flow cytometry, which showed that **A6** did not trigger any of the assayed alterations of the plasma membrane at the concentrations used in the assay (**Fig 4**).

### A6 affects cytosolic Ca^2+^ concentration and mitochondrial functions

**Fig 5:**
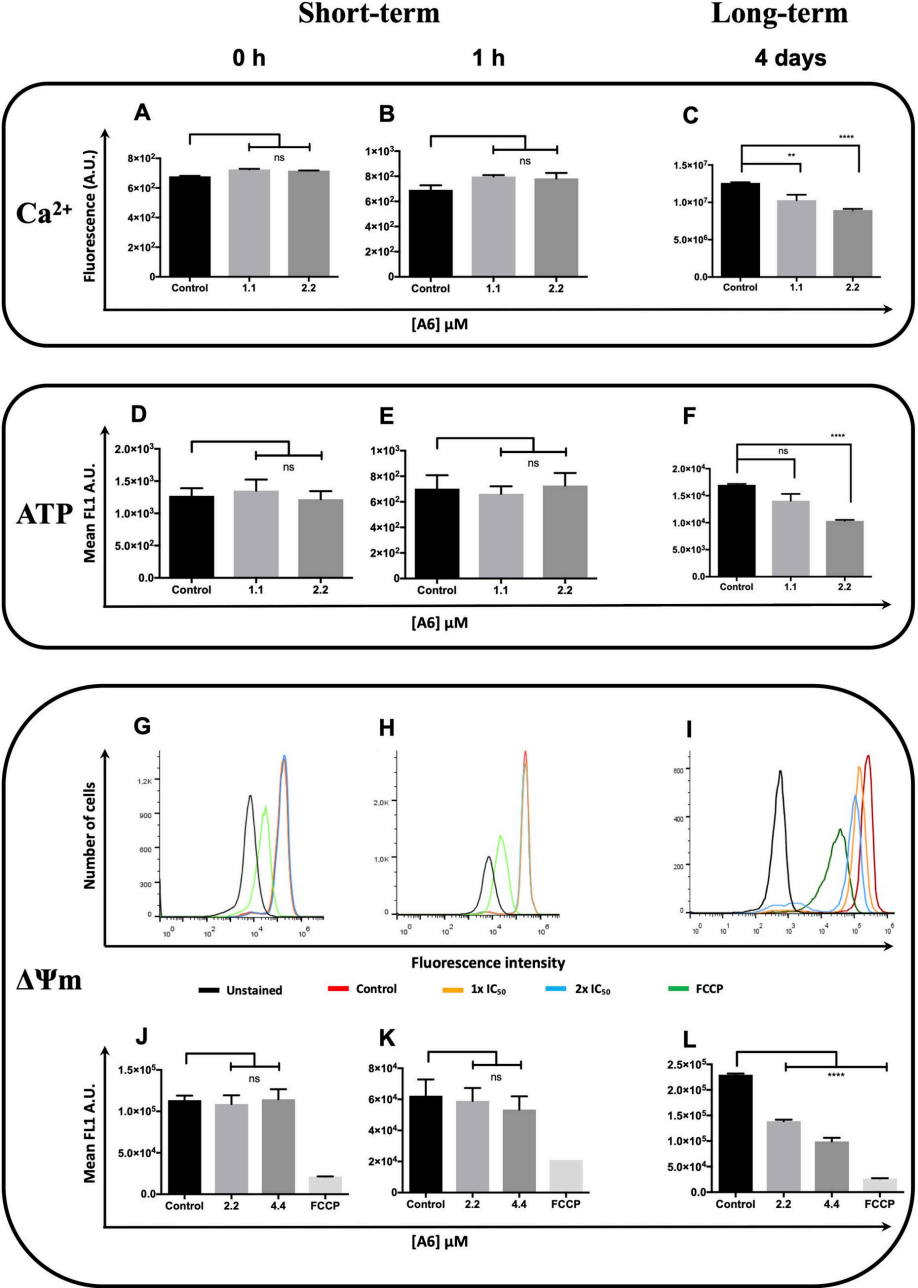
Effect of A6 on the cytosolic Ca^2+^, cytoplasmic ATP content and mitochondrial inner membrane potential of epimastigotes of *T*. *cruzi* at short-term and long-term. The parasites were incubated or not (control) at different times with concentrations corresponding to 1.1, 2.2 or 4.4 μM **A6** (as indicated in each corresponding figure). Quantification of cytosolic Ca^2+^ by fluorometry assay (**λ**_ex_ 490 nm and **λ**_em_ 518 nm): **A**) 0 h, **B**) 1h and **C**) 4 days. Quantification of cytoplasmic ATP levels assessed using a bioluminescent assay (**λ** 570 nm): **D**) 0 h, **E**) 1h and **F**) 4 days. Analysis of mitochondrial inner membrane potential (ΔΨm), the cells were labelled or not (unstained control) with 256 nM Rhodamine 123 (Rho123). In order to obtain a reference value for the parasites with the ΔΨm collapsed, parasites were incubated for 15 min with 10 μM FCCP before the measurements. Each histogram represents the distribution of fluorescence corresponding to ΔΨm for each treated population: **G**) 0 h, **H**) 1h and **I**) 4 days. The peaks of each population were used as a measurement of the average ΔΨm. Values of each bar represent the distances between each peak and that obtained by the reference (FCCP-treated parasites) in fluorescence intensity arbitrary units (A.U.) for each experimental condition: **J**) 0 h, **K**) 1h and **L**) 4 days. Results correspond to mean ± SEM of three independent experiments for each condition and were compared to the control using a *t*-test (**, P < 0.01; ****, P < 0.0001; ns, no significant).

Changes in the intracellular concentration of Ca^2+^ indicate prejudiced ability of epimastigotes to maintain cellular homeostasis. Here we used Fluo-4 AM a dye that is used to label the cytosolic Ca^2+^ [34]. Parasites were incubated with or without (control) 1.1 or 2.2 μM **A6** (corresponding to the 0.5 x and 1 x IC_50_, respectively) at different times, and then the intracellular Ca^2+^ was detected with the probe Fluo-4 AM and quantified by fluorometry. Parasites treated with **A6** did not decrease of the cytosolic Ca^2+^ concentration at short-term assays but exhibited a decrease of the cytosolic Ca^2+^ concentration (18.3% and 28.8% for 0.5x and 1x IC_50_, respectively compared to untreated parasites) at long-term treatment (**Figs 5A, 5B, 5C, and S1**). This result showed that long incubations with **A6** affected the Ca^2+^ homeostasis in the cells.

To determine whether **A6** affects the mitochondrial physiology, we investigated if the treatment triggered changes in the total cellular ATP levels, mitochondrial inner membrane potential (ΔΨ_m_) and ROS production. Epimastigotes were treated with 0.5 x or 1 x IC_50_ of **A6** or left untreated at different times and then, we measured ATP levels using a bioluminescent assay. **A6** did not significantly decrease the ATP levels at short-term treatment or at low concentration but diminished the intracellular ATP content by 39.2% at the IC50 concentration and at long-term treatment when compared to untreated parasites (**Figs 5D, 5E, 5F and S2**). For the assessment of ΔΨ*m*, epimastigotes were treated with **A6** at 1 x or 2 x IC_50_ (or not, control) at different times, stained with Rho123 and analysed by flow cytometry. FCCP was used to obtain a reference fluorescence value in conditions of a complete collapse of ΔΨ*m*. Treated parasites did not decrease in the fluorescence values at short-term analysis but at long-term treatment exhibited a significant decrease in the fluorescence values, showing a dramatic diminution of ΔΨm at both concentrations assessed (**Figs 5G, 5H, 5I, 5J, 5K, 5L and S3**). In addition, we assayed the endogenous production of H_2_O_2_ as a response to treatments with 2.2, 4.4 or 6.6 μM **A6**. We did not observed production of H_2_O_2_ by using the fluorescent probe Amplex Red (**S4 Fig**).

### A6 triggers a cell cycle arrest

**Fig 6:**
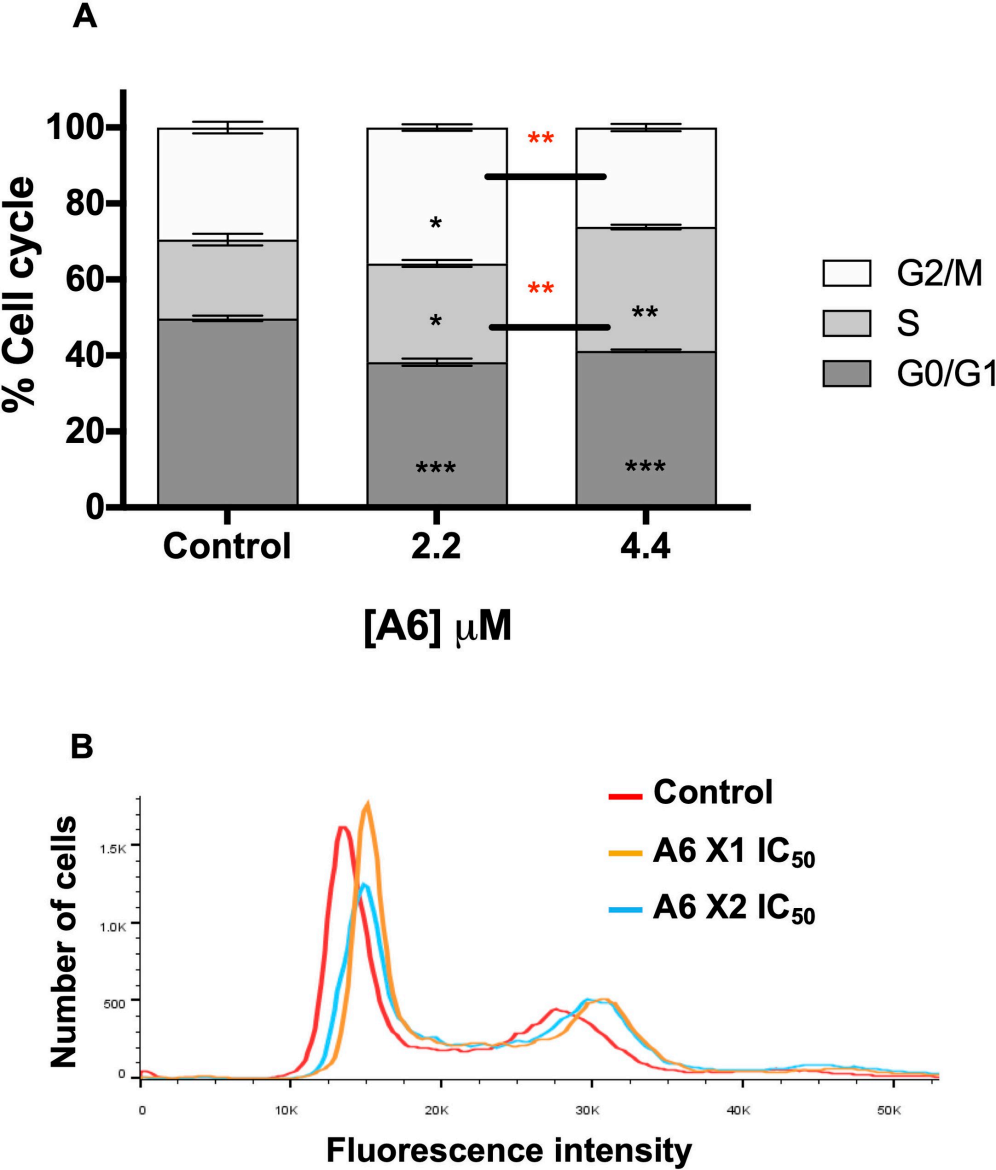
Effect of A6 on the *T*. *cruzi* epimastigotes cell cycle. Cells were treated or not (control) with 2.2 μM or 4.4 μ **A6** for four days (from the lag to the mid-exponential proliferation phase). Then, the cells were washed, treated with RNase A, and stained with propidium iodide, and their DNA content was analysed by cytometry. In total, 50,000 events were analysed for each sample. **A)** Quantification of the labelled cells at each phase of the cell cycle derived from **B)** histograms obtained for the labelled cells in each experimental condition. Results correspond to three independent biological replicas and the values were plotted as the mean ± SEM and compared to the control using a *t*-test (*, P < 0.05; ** P < 0.01; *** P < 0.001). Data correspond to the three independent biological experiments.

As our data suggest that **A6** did not induce cell death by conventional programmed cell-death or necrotic mechanisms, we investigated if the proliferation arrest was due to an interference with the *T*. *cruzi* cell cycle. To verify this hypothesis, we treated the parasites with 2.2 μM and 4.4 μM **A6** (corresponding to 1 x and 2 x IC_50_, respectively), or left them untreated (control) for four days. Then, the cells were labeled with propidium iodide and submitted to cell cycle analysis by flow cytometry. Interestingly, **A6** (both concentrations) triggered a significant decrease of cells in the G_0_/G_1_ phases, and an accumulation of cells in the S phase when compared to the control (**Fig 6**). Additionally, we observed a different alteration between the treatments at both concentrations of the compound. Parasites treated with 4.4 μM **A6**, showed an increase of cells in the S phase and a concomitant decrease in the G_2_/M phase, with no alteration of the G_0_/G_1_ phase, when compared to 2.2 μM **A6** treated parasites.

### A6 selectively inhibits the intracellular cycle of *T*. *cruzi*

**Fig 7:**
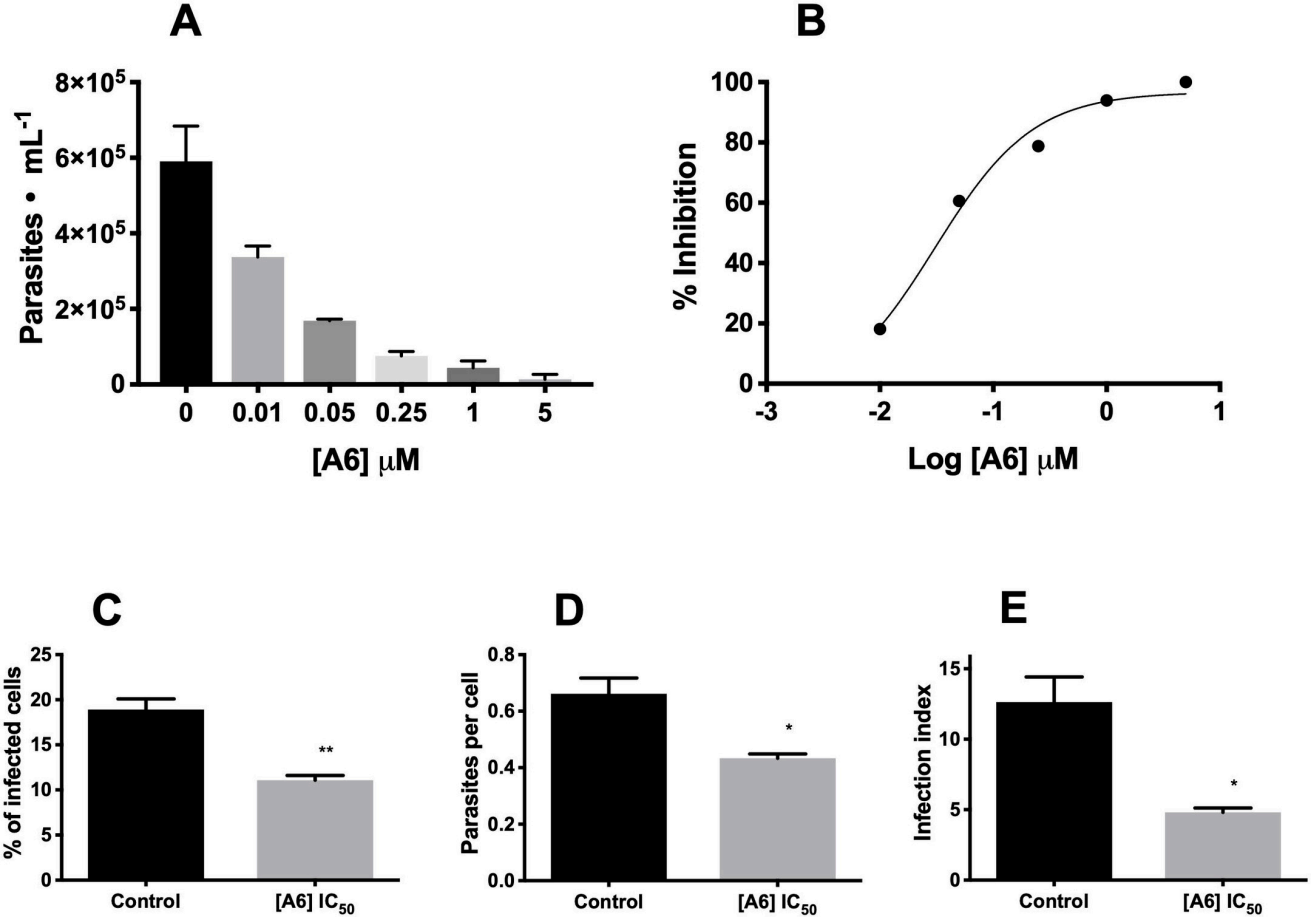
Effect of A6 on the mammalian cell infection by *T*. *cruzi*. The effect of **A6** after infection on CHO-K_1_ cells with trypomastigotes forms. **A)** The released parasites were evaluated by counting the trypomastigotes released from infected mammalian-cell cultures treated (or not, control) with different concentrations of **A6. B)** Data in A) were used to obtain a dose-response curve, which allowed to calculate the IC_50(Tryps)_ = 26.7 ± 3.7 nM. **C)** The effect of **A6** on the successful establishment of the infection was measured as a percent of infected cells at 2 days post-infection. **D)** The number of intracellular amastigotes per infected cell was recorded and **E)** the infection index (percentage of infected cells × the number of parasites per infected cell) was computed for parasites treated or not (control) with 27 nM **A6**. Results correspond to three independent biological replicas and the values were plotted as the mean ± SEM and compared to the control using a *t*-test (*, P < 0.05; **, P < 0.01).

In order to evaluate the effect of A6 on the *T*. *cruzi* mammalian stages, we selected a range of concentrations from 0.01 to 5 μM to obtain the IC_50_ for the trypomastigote release after completion of the infection of a mammalian host-cell. As previously assayed, within this range of concentrations **A6** did not show apparent toxicity for the CHO-K_1_ cells. CHO-K_1_ cells were infected with trypomastigotes and treated or not (control) with different concentrations of **A6**. The trypomastigotes released into the culture medium were counted at the 5^th^ day post-infection. The trypomastigote release exhibited a dose-dependent decrease, with an IC_50 (Tryp)_ of 26.7 ± 3.7 nM for trypomastigotes release (**Fig 7A and 7B**). Based on this value, we computed a selectivity index (*SI*) of 5,170 for **A6**. In order to prove that the selected compound diminished the trypomastigote release due to its effect on the amastigotes proliferation, we treated or not (control) the infected cells with 27 nM **A6** (the concentration corresponding to the IC_50_ for trypomastigotes release) for 2 days after infection. It is worth remarking that during this time, the predominant intracellular stage of the parasite is the amastigote [35]. After fixing and staining the infected cells we quantified the effect of **A6** on the total number of cells, the number of infected cells, and the number of amastigotes per infected cell. **A6** diminished significantly the infected cells by 41% (**Fig 7C**) and the number of parasites per cell by 35% (**Fig 7D**) which diminished the infection index by 62% (**Fig 7E**). Taken together, these results indicate that **A6** interferes the parasites proliferation and/or differentiation during the intracellular infection.

## Discussion

In the present study, we tested a collection of 26 hybrid compounds consisting in conjugates of 7-chloroquinoline and arylamidine, linked through a rigid -O- group. These compounds showed to be DNA binders and to have promising anti-tumoral activities in a previous study [24]. On the basis of an initial screening for an anti-*T*. *cruzi* activity we chose **A6** for further investigating its mechanism of action and its anti-*T*. *cruzi* activity against the clinically relevant parasite forms.

Noteworthy, **A6** did not trigger phosphatidylserine exposure or loss of plasma membrane integrity. When parameters indicating changes in the mitochondrial function were investigated in treated parasites, we observed that in short-term treatments, neither ΔΨm nor ATP levels were affected. However, long-term treatments diminished the ΔΨm and ATP. In addition, short-term treatment with **A6** did not have any detectable effect on cytosolic Ca^2+^. However, long-term treatments triggered its decrease, indicating a profound modification of Ca^2+^ homeostasis along time. The drop in energy levels and mitochondrial membrane potential could be in close relation to the cytosolic Ca^2+^ imbalance. It could even be due to the mobilization of Ca^2+^ towards the mitochondria. It is clear from the magnitude of the effects and its time dependence that the loss of ΔΨm can contribute to the homeostatic imbalance of Ca^2+^, in addition to the disruption of the electron transport chain and consequently lead to failure in the supply of ATP. The main cause of this chain defects could be the inhibition of an essential mitochondrial function or the alteration of the ultrastructure of the mitochondria. Interestingly, not ROS production was detected, as observed for other structurally related compounds. These results are in accordance with previous reports, in which other molecules derived from arylaminoquinolines induced mitochondrial ultrastructural alterations, kinetoplast swelling and vacuolization in *T*. *cruzi* [9,36].

As **A6** and the only other compound that showed anti-*T*. *cruzi* activity (**A4, see Fig 1 and S1 Table)** belong to the group **A** of the collection. We propose that the anti-*T*. *cruzi* effect could be due to the type of chemical substituents, their spatial arrangement and their intramolecular effect on the main molecular structure. Interestingly, imidazolidine derivative, which is an isostere of the substituent of the chemical base **A,** as it is the case of **A6**, seems to be related to the anti-trypanosomal [37–39] and anti-tubercular activities.

**A6** did not trigger the characteristic landmarks of programmed cell death or necrosis, but the cell cycle analysis supports the idea that this compound is a probably trypanostatic rather than trypanocidal. Both, trypanostatic and trypanocidal activities have been previously observed in aromatic diamidines [see for example 18,40]. It was shown that these compounds preferentially bind to the kDNA and interfere with mitochondrial functions and consequently with the proliferation of parasites [18]. This is not surprising, since it has been reported that *T*. *cruzi* can survive for long periods of time without triggering any cell death mechanism after having its proliferation arrested [18,31,41].

A remarkable issue, is that **A6** presented a relevant activity against the mammalian forms of *T*. *cruzi*, illustrated by its effect on intracellular amastigote proliferation and trypomastigote bursting at nanomolar levels. These data, including a very high selectivity index (*SI* of 5,170), are particularly encouraging in order to rank **A6** as a contribution to the identification of novel chemical entities against the Chagas disease.

Although other 7-chloroquinolines derivatives also presented anti-*T*. *cruzi* activities [42,43], this is the first time, to our knowledge, that insight about the mechanism of actions of one of them is proposed.

## Conclusion

To conclude, our work contributes knowledge about the anti-tripanosomatid activity of **A6**, from a previously synthetized 7-chloroquinoline-arylamidine collection, which showed remarkable *in vitro* antiparasitic effects. Our data indicate that chloroquinoline derivatives substituted with aromatic imidazolines, such as **A6,** could be used as an interesting lead compound for the development of new and better chemotherapy against *T*. *cruzi*.

## Supporting information

S1 Table Percentage of inhibition obtained at 5 μM for each compound. (PDF)

S1 Fig Effect of A6 on the cytosolic Ca^2+^ of epimastigotes of *T*. *cruzi* at short-term and long-term. The parasites were incubated or not (control) at different times with concentrations corresponding to 1.1 or 2.2 μM **A6**. Quantification of cytosolic Ca^2+^ by fluorometry assay (**λ**_ex_ 490 nm and **λ**_em_ 518 nm). Results correspond to mean ± SEM of three independent experiments for each condition and were compared to the control using a *t*-test (**, P < 0.01; ****, P < 0.0001; ns, no significant).
(TIFF)

S2 Fig Effect of A6 on the cytoplasmic ATP content of epimastigotes of *T*. *cruzi* at short-term and long-term. The parasites were incubated or not (control) at different times with concentrations corresponding to 1.1 or 2.2 μM **A6**. Quantification of cytoplasmic ATP levels assessed using a bioluminescent assay (**λ** 570 nm). Results correspond to mean ± SEM of three independent experiments for each condition and were compared to the control using a *t*-test (****, P < 0.0001; ns, no significant).
(TIFF)

S3 Fig Effect of A6 on the mitochondrial inner membrane potential (ΔΨm) of epimastigotes of *T*. *cruzi* at short-term and long-term. The parasites were incubated or not (control) at different times with concentrations corresponding to 2.2 or 4.4 μM **A6**. Analysis of ΔΨm, the cells were labelled or not (unstained control) with 256 nM Rhodamine 123 (Rho123). In order to obtain a reference value for the parasites with the ΔΨm collapsed, parasites were incubated for 15 min with 10 μM FCCP before the measurements. **Panel A**: each histogram represents the distribution of fluorescence corresponding to ΔΨm for each treated population. **Panel B**: the peaks of each population were used as a measurement of the average ΔΨm. Values of each bar represent the distances between each peak and that obtained by the reference (FCCP-treated parasites) in fluorescence intensity arbitrary units (A.U.) for each experimental condition. Results correspond to mean ± SEM of three independent experiments for each condition and were compared to the control using a *t*-test (****, P < 0.0001; ns, no significant).
(TIFF)

S4 Fig Analysis of the ROS generation by epimastigotes of *T*. *cruzi* treated with A6. H_2_O_2_ production was measured using intact parasites in exponential proliferation phase treated or not with **A6.** The cells were added to the MCR buffer containing 5 μM Amplex red and 0.01 μg/mL of Horse Radish Peroxidase. Then, three pulses of **A6** were sequentially added to record the effect of a gradually increased concentration of the compound. Each pulse increased the A6 concentration by 2.2 μM). The rates of H_2_O_2_ production were measured by fluorometry in a high-resolution oxygraph equipped with a fluorometer device. **Insert:** The slope variation (representing the H_2_O_2_ production rate) after each addition of **A6** were computed and recorded. Each condition was run in three biological independent experiments and the values correspond to the mean ± SEM and were compared to the control by using a *t*-test (P < 0.05).
(TIFF)
