# Supplementary material for: An aromatic imidazoline derived from chloroquinoline triggers cell cycle arrest and inhibits with high selectivity the Trypanosoma cruzi mammalian host-cells infection
Source: PLoS Negl Trop Dis. 2021 Nov 29;15(11):e0009994. doi: 10.1371/journal.pntd.0009994 (PMC8659321; doi:10.1371/journal.pntd.0009994)
Supplement: S1 Table — (PDF) [file pntd.0009994.s001.pdf]

S1 Table Percentage of inhibition obtained at 5 μM for each compound

Compound	% inhibition*
A1	NI[a]
A2	NI
A3	19.2 ± 3.9
A4	31.4 ± 2.4
A5	17.6 ± 3.1
A6	89.4 ± 1.8
A7	NI
B2	NI
B3	NI
B4	NI
B5	NI
B6	NI
B7	NI
C1	0.7 ± 4.9
C3	NI
C4	NI
C5	NI
C6	NI
C7	NI
D1	NI
D2	NI
D3	NI
D4	NI
D5	NI
D6	NI
D7	NI
C+[b]	82.1 ± 11.8

[a]NI = no inhibition

[b]C+ = Benznidazole at 20 μM

*= mean ± SD at the mid-exponential phase (4th day)
